# Case Report: Hematologic Recovery Following Stereotactic Ablative Radiotherapy in a Patient With Early-Stage Non-Small Cell Lung Cancer and Paraneoplastic Myelofibrosis

**DOI:** 10.3389/fonc.2022.842620

**Published:** 2022-02-24

**Authors:** Lindsey Sloan, Rakhi P. Naik, Kavita Umrau, Rena Ruiyu Xian, Kristen A. Marrone, Khinh Ranh Voong

**Affiliations:** ^1^ Department of Radiation Oncology, University of Minnesota Medical School, Minneapolis, MN, United States; ^2^ Department of Radiation Oncology and Molecular Radiation Sciences, Johns Hopkins School of Medicine, Baltimore, MD, United States; ^3^ Department of Hematology, Johns Hopkins School of Medicine, Baltimore, MD, United States; ^4^ Department of Pathology, Johns Hopkins School of Medicine, Baltimore, MD, United States; ^5^ Department of Oncology, Johns Hopkins School of Medicine, Baltimore, MD, United States

**Keywords:** NSCLC, SAbR, paraneoplastic, case, report

## Abstract

Herein, we report the first case presentation of paraneoplastic myelofibrosis associated with cancer. Paraneoplastic syndromes occur in some patients with thoracic malignancies; however, myelofibrosis is not commonly seen in non-small cell lung cancer (NSCLC). We report a case of myelofibrosis in a patient with a new diagnosis of NSCLC that resolved after stereotactic ablative radiotherapy (SABR). In conclusion, NSCLC may evoke unexpected systemic effects that resolve with treatment.

## Introduction

Paraneoplastic syndromes are extreme examples of the systemic influence of cancer. They are defined as a constellation of spatially distant signs and/or symptoms of a primary cancer (1). Paraneoplastic syndromes are believed to occur infrequently; however, their prevalence may be underestimated due to underreporting. Small cohort studies have reported the incidence of paraneoplastic syndromes to be 11%–16% in lung cancer patients (2–4). Paraneoplastic disorders are diagnoses of exclusion; however, diagnostic criteria have been developed for some syndromes with available specialized testing (5, 6). These syndromes occur due to two main mechanisms: the generation of physiologic antibodies directed against a tumor epitope that cross reacts with normal tissue (neurologic paraneoplastic syndromes) and abhorrent production of a functional soluble factor ectopically produced by tumor cells (endocrine paraneoplastic syndromes) (7). Antibodies against neuronal/glial tissue are most commonly appreciated within neurologic paraneoplastic syndromes, as in paraneoplastic cerebellar degeneration (anti-Yo or anti-Hu antibodies) (8) and Lambert-Eaton myasthenia syndrome (anti-P/Q type voltage-gated calcium channel antibodies) (9). Syndromes characterized by endocrine or metabolic disturbances may involve production of inappropriate or supra-physiologic levels of a protein (hypercalcemia *via* parathyroid or parathyroid hormone-related peptide) (10). Specific treatment algorithms for some paraneoplastic syndromes do exist, especially in antibody-mediated syndromes and those requiring urgent medical intervention (10). Management, however, of the underlying cancer is often considered the first step in treatment (6).

Out of all thoracic malignancies, paraneoplastic syndromes such as Cushing’s syndrome and syndrome of inappropriate antidiuretic hormone secretion are most commonly associated with small cell lung cancer (6). In non-small cell lung cancer (NSCLC), end organ damage to nervous and hormone-responsive tissue by paraneoplastic syndromes is most often reported, but involvement of the hematopoietic system also occurs infrequently (2, 3). A recent Danish registry cohort study estimated the incidence of hematologic paraneoplastic syndromes in NSCLC to be 1.9% (2). Paraneoplastic syndromes causing anemia are rare. In terms of thoracic cancer-related anemia, pure red cell aplasia in patients with thymoma is one of the more commonly discussed blood-related paraneoplastic syndromes (11). Here, we report the first known case of paraneoplastic myelofibrosis in lung cancer.

## Case Description

A 69-year-old non-smoking patient was seen in our thoracic multidisciplinary clinic to discuss management options for his newly diagnosed Stage 1A3, T1cN0M0 lung adenocarcinoma (programmed death ligand 1 score <1%). During this visit, he was noticeably unwell and reported new-onset dyspnea.

**Figure 1:**
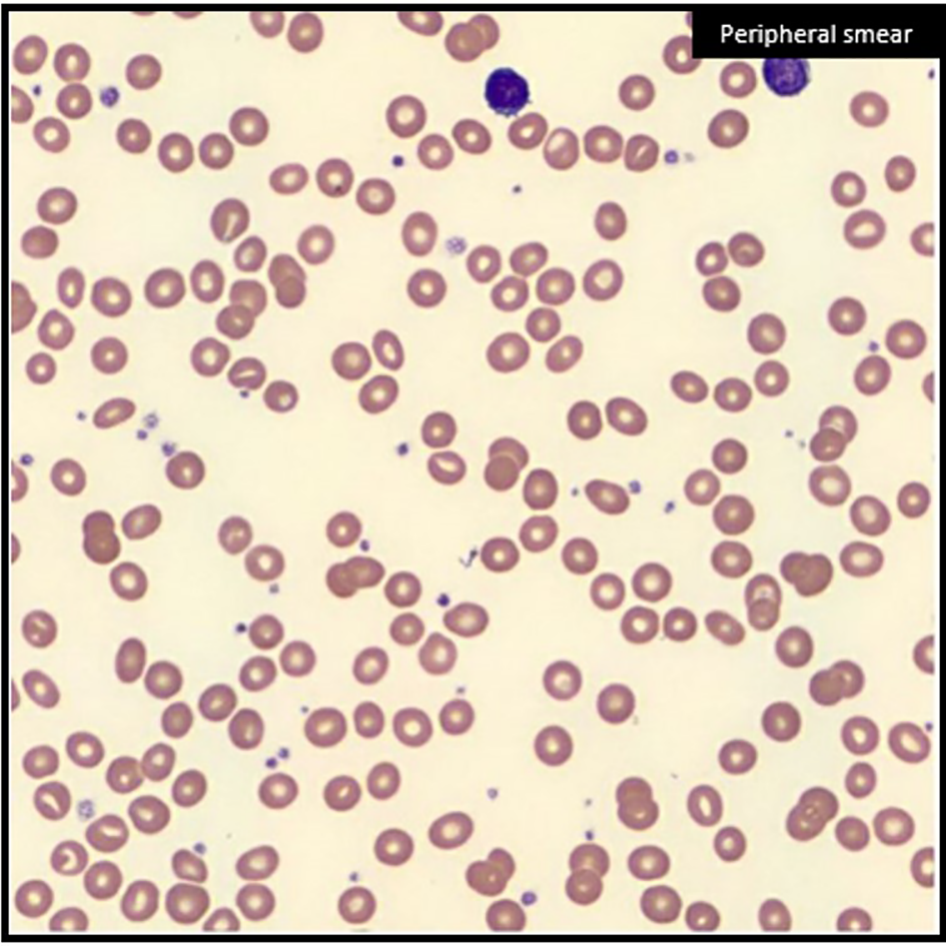
Normocytic anemia on blood smear. Peripheral blood smear at the time initial diagnosis with findings of normocytic anemia with reticulocytopenia, but notable absence of teardrop forms (dacrocytes).

Upon admission, he was found to have a profound normocytic anemia on complete blood count (hemoglobin: 4.2 g/dl, low; reticulocyte percent: 0.3%, low; platelets/white blood cells: within normal limits) Peripheral blood smear (**Figure 1**) depicted red blood cells with a normal structure with a notable absence of teardrop forms. Further emergent workup identified a lactate dehydrogenase (LDH) of 214 U/l (high), haptoglobin of 205 mg/dl (high), iron of 186 μg/dl (high), transferrin 150 mg/dl (low), total iron binding capacity of 188 μg/dl (low), and percent saturation of 99% (high). Additional laboratory tests were not suggestive of hemolysis despite mildly elevated LDH and haptoglobin: total bilirubin 0.7 mg/dl and alanine aminotransferase (ALT) 28 U/l. His anemia required transfusion of 5 units of blood. Trauma and gastrointestinal sources of blood loss were ruled out.

**Figure 2:**
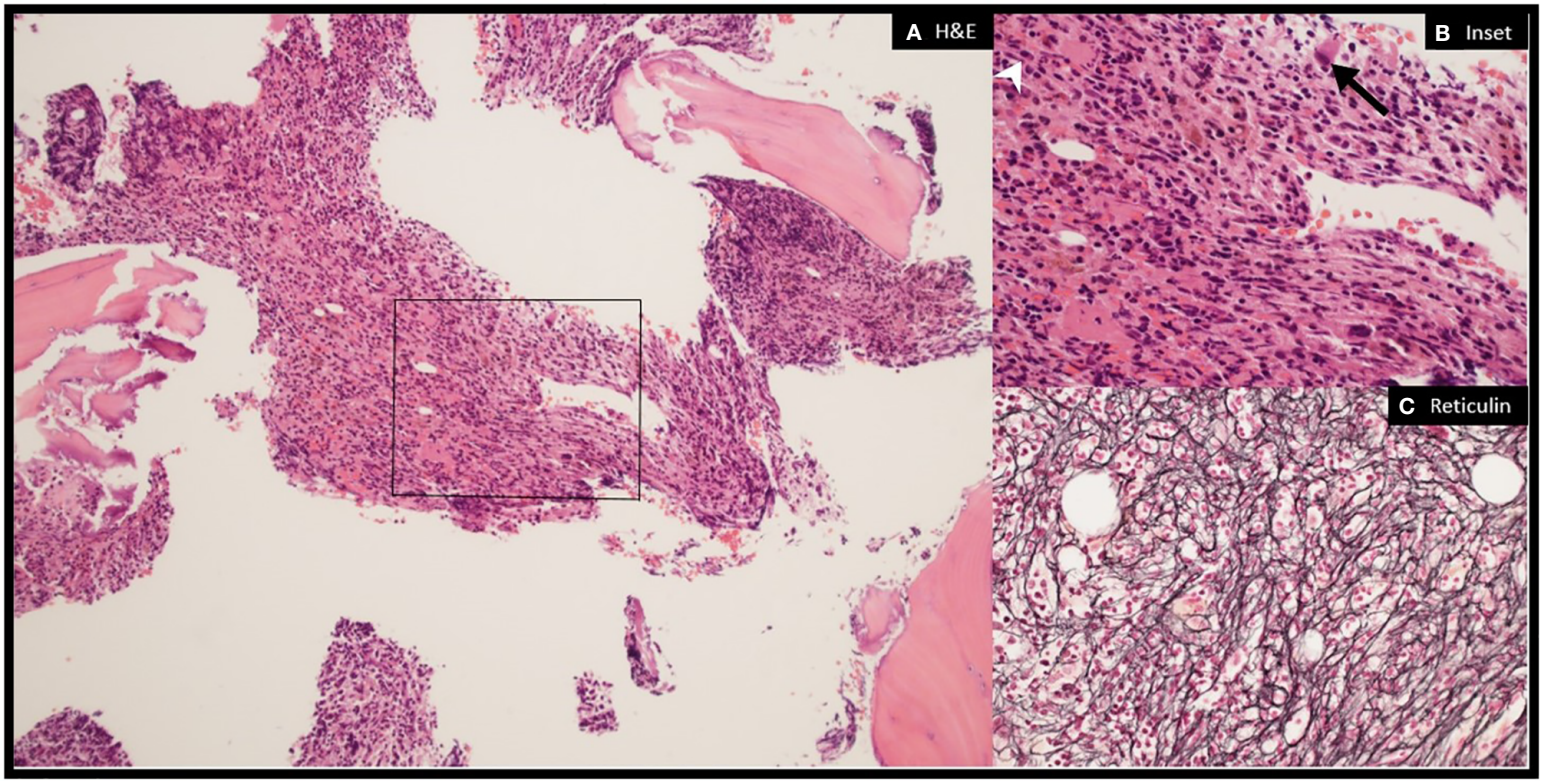
Myelofibrosis on bone marrow biopsy. Bone marrow biopsy showing markedly hypercellular and fibrotic marrow with trilineage hematopoiesis. The megakaryocytes are adequate in number but show atypical forms including, large hypo-lobated cells (black arrow), cells with hyperchromatic nuclei, as well as small mono-lobated forms (white arrowhead) **(A)** (×10) and **(B)** (×40): H&E). Reticulin stain demonstrates moderate–to–severe reticulin fibrosis, MF-2 to MF-3 based on WHO grading **(C)** (×40): reticulin).

Hematology was consulted. The patient was not found to have splenomegaly on exam. Additional laboratory workup of the patient’s anemia revealed a ferritin of 1395 ng/dl (high), reticulocyte count of 7.2 K/cu mm 3% (low), serum folate of 6.6 ng/ml (normal), vitamin B12 of 427 pg/ml (normal), and serum erythropoietin of 709.3 mIU/ml (high). Molecular diagnostic and antibody testing for viral infections were negative. ANA was positive (1:360). Fluorescence *in situ* hybridization (FISH) for adult acute myeloid leukemia (AML) and myelodysplastic syndromes (MDS) identified no mutations using probes 5p15.2 (D5S23, D5S721), 5q31 (EGR1), 7cen(D7Z1), 7q31 (D7S522), 8cen (D8Z2), 11q23 (MLL), 20q12 (D20S108), and 20q13.12 (D20S150). The patient’s karyotype was normal 46,XY (12). Next-generation sequencing (NGS), comprising a panel of 87 genes, was performed and was negative for common driver mutations of hematologic cancers including JAK2 and MPL. Bone marrow aspirate was performed (**Figure 2**). Flow cytometry identified a 1.5% B cell clone of unknown significance with a chronic lymphocytic leukemia (CLL) phenotype. Ultimately, the patient was diagnosed with myelofibrosis of paraneoplastic or autoimmune etiology. He continued to have refractory and symptomatic anemia with persistent reticulocytopenia despite treatment with 2 weeks of oral steroids.

**Figure 3:**
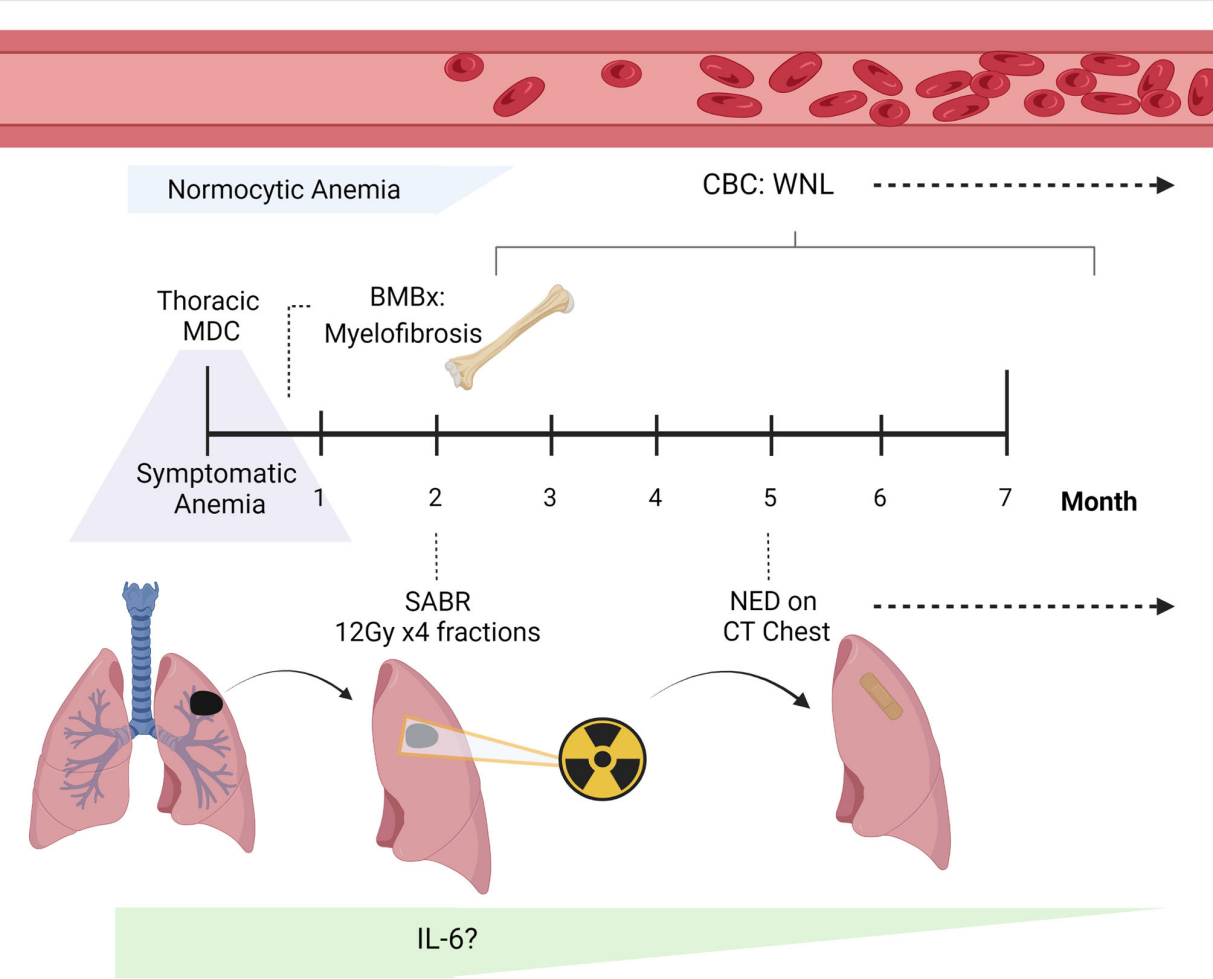
Clinical timeline of the case report. The case report of our patient with a lung adenocarcinoma who was found to have new-onset dyspnea during his visit to our Thoracic Multi-disciplinary Clinic (MDC) for treatment recommendations. Because he was noticeably unwell at this appointment, he underwent urgent evaluation and was found to have normocytic anemia. The patient’s workup was significant for myelofibrosis on bone marrow biopsy (BMBx). He was found to have steroid-refractory disease, and his anemia persisted on complete blood count (CBC). Following treatment of his lung cancer with stereotactic ablative radiotherapy (SABR), the patient had no evidence of disease (NED) on CT chest. Unexpectedly, following SABR, his CBC normalized and blood counts returned to values within normal limits (WNL). No additional treatment was needed for his symptomatic anemia over 1 year after SABR, and he continues to be surveilled for his lung cancer. This figure was created with BioRender.com.

The care plan was to proceed with curative management of the patient’s non-small cell lung cancer (NSCLC) with stereotactic ablative radiotherapy (SABR), after which treatment of his transfusion-dependent anemia with rituximab would be considered. The patient received SABR (48 Gy in 4 fractions) for his Stage 1A3 NSCLC without adverse or unexpected events. Within 1 week of radiotherapy (RT), the patient’s reticulocyte count increased to 5.1% (above normal limits) and hemoglobin increased from 7.9 to 8.1 g/dl. He did not experience any side effects from SABR. He had normalization of his hemoglobin for an extended 14 months after SABR (16 g/dl) without need for interval hematologic intervention. The patient developed relapse of his autoimmune fibrosis in the setting of vaccination against the coronavirus disease 2019 (COVID-19) and redeveloped a transfusion-dependent anemia. Positron-emission tomography at the time of redevelopment of his transfusion-dependent anemia showed no evidence of lung cancer recurrence. Repeat bone-marrow biopsy showed hypercellular bone marrow with mildly increased reticulin fibrosis. The persistence of a small CLL clone was detected and for which he received rituximab without response. This patient remains transfusion dependent. The patient provided informed consent for this case report, and the timeline of his hematopoietic response after completion of SABR is depicted in **Figure 3**.

## Discussion

The patient initially presented with severe, likely acute, anemia at his consultation for radiation therapy for an early-stage NSCLC. His hemoglobin was found to be 4 g/dl, requiring urgent transfusion. Further workup of his anemia ruled out hemolysis, as he had a mildly elevated LDH and haptoglobin in the presence of a normal total bilirubin and ALT with no teardrop-shaped red blood cells on peripheral blood smear. Although teardrop forms are typically identified on bone marrow fibrosis, we suspect the lack of dacrocytes seen here likely relates to the acute nature of the patient’s clinical presentation. This would be analogous to acute panmyelosis with myelofibrosis, a rapidly progressive disease, which shows severe marrow fibrosis without circulating teardrop cells. Since the lifespan of a red blood cell is 120 days and this patient’s symptoms occurred much more quickly, we suspect that our assessment of the blood smear was prior to the development of dacrocytes. Severe anemia, as seen with our patient’s hemoglobin of 4 g/dl, from chronic myelofibrosis is usually accompanied by other blood abnormalities and splenomegaly. Splenomegaly was not seen. However, cases of acute myelofibrosis and autoimmune myelofibrosis are often not accompanied by splenomegaly, and autoimmune myelofibrosis, in particular, may not be associated with pancytopenia. In this case, the red cell line was primarily affected.

Although paraneoplastic myelofibrosis has never been reported to our knowledge, abnormal hematologic findings in cancer patients are common. These alterations may be explained by the use of cancer-directed systemic therapies, infection, a chronic disease state, the presence of bone marrow metastases, or steroid use (13). When paraneoplastic syndromes are responsible for hematologic finding associated with cancer, they are usually increases in a circulating cell type (e.g., leukocytes, erythrocytes, thrombocytes) and less commonly a reduction in a cell population (14). Castello and colleagues were able to study cancer patients with under-functioning bone marrow in detail (15). Out of a group of mixed histology solid cancer patients without known metastatic bone marrow involvement, they found that a decrease in multiple hematopoietic cell lineages including erythrocytes, granulocytes, and megakaryocytes occurred most commonly in patients with gastrointestinal cancers, followed by those with lung cancers (15).

**Table 1** summarizes the available literature regarding hematologic paraneoplastic syndromes associated with NSCLC. Hypereosinophilia and leukemoid reactions, or massive increases in white blood cells with a predominance of neutrophils, are the most commonly reported hematopoietic paraneoplastic syndromes in NSCLC. Hypereosinophilia is defined as more than 1,500 eosinophils per μL of blood (17); leukemoid reactions are characterized by more than 50,000 white blood cells per μL of blood (22). A link between a growth factor that supports the maturation of eosinophil progenitor cells, interleukin (IL)-5, and hypereosinophilia is known (24, 27). In support of the chief role for tumor cells in some paraneoplastic syndromes, one case report found high levels of systemic IL-5 to fall following the resection of a NSCLC (27). Similar to the connection of IL-5 and hypereosinophilia, overproduction of granulocyte-colony stimulating factor (G-CSF) has been attributed as the cause of paraneoplastic leukemoid relations in some patients (13). One case report found a robust expression of G-CSF to explain the increase in circulating leukocytes in conjunction with the marrow-active cytokine, interleukin (IL)-6 (22). Unfortunately, both hypereosinophilia and leukemoid reactions are associated with poor outcomes in cancer patients (13, 26). This suggests that further understanding of paraneoplastic mechanisms, which may occur subclinically in more patients than are formally diagnosed with paraneoplastic syndromes, could be important to the development of future novel anti-cancer therapies and treatment adjuvants.

**Table 1:** Hematologic paraneoplastic syndromes reported in non-small cell lung cancer patients.

Year	First author	Study type	Lung cancer histology	Hematologic paraneoplastic finding
2021	Wehbe (16)	Case report	Adenocarcinoma	Hypereosinophilia
2020	Akkad (17)	Case report	Adenocarcinoma	Hypereosinophilia; leukemoid reaction
2020	Chahine (18)	Case report	Adenocarcinoma	Leukemoid reaction
2019	Mouhayyar (19)	Case report	Adenocarcinoma	Hypereosinophilia
2018	Abughanimeh (20)	Case report	Adenocarcinoma	Hypereosinophilia
2016	Yu (21)	Case report	Adenocarcinoma	Anemia; thrombocytopenia
2015	McCoach (22)	Case report	Adenocarcinoma	Leukemoid reaction
2014	Youssef (23)	Case report	Squamous cell carcinoma	Hypereosinophilia
2013	Lo (24)	Case report	Adenocarcinoma	Hypereosinophilia
2012	Riesenberg (25)	Case report	Adenocarcinoma	Leukemoid reaction
2011	Verstraeten (26)	Case report	Non-small cell lung cancer^a^	Hypereosinophilia
2007	Pandit (27)	Case report	Large cell carcinoma	Hypereosinophilia
2001	Kasuga (13)	Case series	32 Non-small cell lung cancer (14 adenocarcinoma; 12 squamous cell carcinoma; 6 large cell carcinoma)	Leukocytosis
1992	Sans-Sabrafen (28)	Case series	2 adenocarcinomas	Refractory anemia with ring sideroblasts
1984	Raz (14)	Case series	3 non-small cell lung cancer (2 adenocarcinoma, 2 squamous cell carcinoma)	Myelodysplasia

a This report was of a patient with non-small cell lung cancer, without additional histologic subclassification. General lab abnormality definitions: Leukemoid reaction >50,000 cells/μl; leukocytosis >10,000 cells/μl; hypereosinophilia >1,500 cells/μl.

Of note, reports of cell changes due to autoimmune antibody production were not included in **Table 1**. We acknowledge that paraneoplastic syndromes may also be associated with expansion of specific peripheral lymphocyte populations. Antibody-mediated myelofibrosis remains in the differential diagnosis for our patient, especially in the setting of (1) his normal lymphocyte counts on his initial CBC test and (2) his persistent anemia despite trial of rituximab for the small population of CLL cells found on his repeat bone marrow biopsy over 1 year later. Further, because we did not perform immunoassays to detect bone marrow-reactive antibodies, autoimmune etiologies of our patient’s anemia cannot be excluded despite steroids not being effective in correcting his anemia.

Although initially it appeared that our patient had two independent disease processes, myelofibrosis and NSCLC, the complete resolution of the patient’s anemia after SABR suggested a connected pathophysiology. Primary myelofibrosis has been associated with excess circulating serum cytokines including, IL-6 (11), which is also highly expressed by NSCLC tumor cells (12). IL-6 has been reported to be involved in the pathophysiology of multiple paraneoplastic syndromes, including the aforementioned leukemoid reactions (29–31). As cytokines have been reported to be responsible for some paraneoplastic hematopoietic syndromes in patients with NSCLC, as described above (32), we propose that one potential mechanism to explain these findings could involve systemic IL-6 levels. We hypothesize that tumor cells secreting IL-6 may have been responsible for the development of myelofibrosis in this patient (**Figure 3**). Further, these tumor cells were destroyed by ablative radiation which subsequently resulted in normal bone marrow function and red blood cell production. We are limited in the retrospective nature of this report as we cannot evaluate for the level of IL-6 prior to and after receipt of SABR; however, clearly SABR was the only therapeutic intervention that occurred prior to this patient’s hematopoietic recovery. As conventionally fractionated radiation has been shown to have an effect on serum IL-6 (33), this mechanism is likely to be much more complex. A translational study of the biological effects of even relatively small volumes of ablated tissue, as seen in SABR for early lung cancers, is needed. Finally, further hematologic cytokine level evaluation will be needed if there is future clinical concern of tumor progression.

The recurrence of the patient’s anemia following his first COVID-19 vaccination is an interesting observation. Temporally, the relapse of the patient’s condition did appear to be related to the COVID-19 vaccine. We would assume that this is immune-mediated, although we cannot speculate on the exact mechanism. Certainly, his underlying B cell clone with CLL immunophenotype likely predisposed to immune complications. Other autoimmune phenomena such as immune thrombocytopenia purpura (ITP) certainly are known to occur with COVID infection and with COVID vaccination.

In conclusion, this report is the first to identify resolution of a myelofibrosis associated with cancer. The anemia found in our patient was due to a myelofibrotic process of the bone marrow caused by the patient’s small, but biologically active, lung cancer. The patient we describe experienced hematologic recovery after treatment his NSCLC with SABR. One possible cytokine responsible may be IL-6.

## Methods

### Literature Search

PubMed was queried for available full-text articles printed in the English language using the terms “paraneoplastic non-small cell lung cancer” and “paraneoplastic syndrome lung adenocarcinoma”. Published cases reporting abnormalities in the quantity of common cellular blood components due to a paraneoplastic syndrome from NSCLC were reviewed and are shown in **Table 1**.

### Patient Perspective

The patient provided written consent for the use of his clinical case for the education purposes of this article. The patient continues to undergo treatment for CLL and management of his recurrent anemia.

## Data Availability Statement

The raw data supporting the conclusions of this article will be made available by the authors, without undue reservation.

## Ethics Statement

Ethical review and approval were not required for the study on human participants in accordance with the local legislation and institutional requirements. The patients/participants provided their written informed consent to participate in this study. Written informed consent was obtained from the individual(s) for the publication of any potentially identifiable images or data included in this article.

## Author Contributions

All authors were involved in the care of the patient, as well as the writing and review of the manuscript. LS was directly involved in literature review. RPN provided hematology expertise and the case images. KAM was involved in oncologic care of this patient. KU and RRX were involved in pathologic diagnosis of the patient and figure preparation. KRV oversaw radiation oncology care. All authors contributed to the article and approved the submitted version.

## Funding

Open access fees were paid by KRV from gift funds.

## Conflict of Interest

KAM is on the advisory boards of Amgen, AstraZeneca, and Mirati Therapeutics. KRV disclosures include the following: honorarium—Physicians’ Education Resource, Consultant—AstraZeneca.

The remaining authors declare that the research was conducted in the absence of any commercial or financial relationships that could be construed as a potential conflict of interest.

## Publisher’s Note

All claims expressed in this article are solely those of the authors and do not necessarily represent those of their affiliated organizations, or those of the publisher, the editors and the reviewers. Any product that may be evaluated in this article, or claim that may be made by its manufacturer, is not guaranteed or endorsed by the publisher.
